# Curcumin-Artesunate Based Polymeric Nanoparticle; Antiplasmodial and Toxicological Evaluation in Murine Model

**DOI:** 10.3389/fphar.2018.00562

**Published:** 2018-05-30

**Authors:** Oyetunde Oyeyemi, Olajumoke Morenkeji, Funmilayo Afolayan, Kabiru Dauda, Zulaikha Busari, Jairam Meena, Amulya Panda

**Affiliations:** ^1^Department of Biological Sciences, University of Medical Sciences, Ondo City, Nigeria; ^2^Product Development Cell, National Institute of Immunology, New Delhi, India; ^3^Department of Zoology, University of Ibadan, Ibadan, Nigeria

**Keywords:** polymeric nanoparticles, curcumin, artesunate, antimalarial, safety

## Abstract

Mainstay chemotherapy for malaria is often faced with the problem of instability and poor bio-distribution thus resulting in impaired pharmacokinetics. Nanomedicine has been acclaimed for its success in drug delivery and improved efficacy. The aim of the study was to assess the antiplasmodial efficacy and safety of curcumin-artesunate co-entrapped nanoparticle in mice model. Curcumin (C) and artesunate (A) were loaded in poly (d,l-lactic-*co*-glycolic acid) (PLGA) using solvent evaporation from oil-in-water single emulsion method. The nanoparticle formed was characterized for size, polydispersity index (PDI), zeta potential, and entrapment efficiency. The *in vitro* release of the drug was also determined. The *in vivo* antiplasmodial activity of CA-PLGA nanoparticle was tested on *Plasmodium berghei* at 5 and 10 mg/kg doses. The drug efficacy was determined at day 5 and 8. Hematological and hepatic toxicity assays were performed. The mean particle size of drug entrapped PLGA-nanoformulation was 251.1 ± 12.6 nm. The drug entrapment efficiency was 22.3 ± 0.4%. There was a sustained drug release from PLGA for 7 days. The percentage suppression of *P. berghei* was consistently significantly higher in CA-PLGA 5 mg/kg at day 5 (79.0%) and day 8 (72.5%) than the corresponding values 65.3 and 64.2% in the positive control group (*p* < 0.05). Aspartate aminotransferase (AST) was significantly lower in mice exposed to 5 mg/kg (42.0 ± 0.0 U/L) and 10 mg/kg (39.5 ± 3.5 U/L) nanotized CA-PLGA compared with the negative control (45.0 ± 4.0 U/L) (*p* < 0.05). Although alanine aminotransferase (ALT) was lower in nanotized CA-PLGA, the variation was not significant compared with the negative control (*p* > 0.05). No significant difference in the mean values of the different blood parameters in all exposed groups with the exception of platelets which were significantly higher in the positive control group. A simple method of dual entrapment of curcumin and artesunate with better antiplasmodial efficacy and low toxicity has been synthesized.

## Introduction

Malaria is still a major cause of mortality and morbidity in sub-Saharan Africa especially among pregnant women and children <5 years of age (World Health Organization, 2011). Considerable efforts have been made toward malaria eradication in endemic areas. Chemotherapy has played an important role in the fight against the disease. Artemisinin and its semi-synthetic derivatives such as artemether, hydroartemisinin, and artesunate are the most important antimalarial drugs and have been implemented in combination therapies (ACTs) for enhanced activities. Despite the initial success acclaimed for combination therapy, failure has been recorded in some endemic countries (Dondorp et al., 2009; Lim et al., 2009). This resistance may be partly due to some biopharmaceutical issues associated with artemisinin-based therapies and which have been noted also for artesunate; a derivative from the parent compound. Notable among these untoward properties of these compounds that result in poor pharmacokinetics are poor bioavailability, low solubility, and instability (Isacchi et al., 2012; Meng et al., 2014).

Curcumin from turmeric due to its short half-life has similar pharmacokinetic properties with artemisinin (Nandakumar et al., 2006) and has been proposed to offer optimum mutual protection against falciparum malaria (Nosten and White, 2007). Curcumin therapeutic potency was improved in combination with artemisinin-based derivatives in preventing parasite recrudescence in mice infected with *Plasmodium berghei* for 24 h (Nandakumar et al., 2006). This potential application of curcumin in combination therapy is especially favored by its relative abundance and cost effectiveness (Reddy et al., 2005). Curcumin also suffers similar set-back as artemisinin and derivatives as it is poorly soluble and bioavailable (Anand et al., 2007).

Delivery systems such as poly (d,l-lactic-*co*-glycolic acid) (PLGA) have been widely exploited in drug delivery to target intracellular pathogens like malaria parasites (Nayaka et al., 2010; Busari et al., 2017; Dauda et al., 2017). Besides, PLGA was successfully used to deliver antihelminthic and anticancer drugs (Nguyen et al., 2015; Oyeyemi et al., 2018). The use of PLGA delivery system poses further advantage in that it produces nanoparticles which are less toxic than the corresponding free drugs (Dauda et al., 2017). While preclinical studies on antiplasmodial efficacy of curcumin combination therapy with one or more of the artemisinin derivatives are becoming more common, information on their corresponding nanotized formulation is still few. Curcumin and artemisinin loaded PEGylated liposomal nanoparticle was reported to significantly suppress *P. berghei* than the corresponding parent free drugs (Isacchi et al., 2012). In a bid to further unravel the antiplasmodial potential of nanotized curcumin and artemisinin-based derivatives nanoparticle, we formulated a curcumin and artesunate loaded PLGA nanoparticle to offer a novel therapeutic approach for malaria treatment. The aim of this study was to evaluate the antiplasmodial efficacy and safety of a novel curcumin-artesunate based PLGA nanoparticle.

## Materials and methods

### Materials

Curcumin (from *Curcuma longa* Linn), artesunate (from *Artemisia annua*), polyvinyl alcohol (PVA) (M_W_ = 30–70 kDa), D-mannitol were purchased from Sigma-Aldrich (St. Louis, MO, USA). Poly (d,l-lactic-*co*-glycolic acid) (intrinsic viscosity η = 0.41 dL/g, copolymer ratio 50:50, 45 kDa) was purchased from Purac Biochem, Holland. Dichloromethane and acetone were procured from Merck Serono Ltd. Water purified by Milli-Q_plus_ system from Millipore (MQ water) was used. All other chemicals were of analytical grade.

### Preparation of nanoparticle

Curcumin-artesunate PLGA entrapped nanoparticle (CA-PLGA) was formulated by solvent evaporation from oil-in-water single emulsion method as described by Busari et al. (2017). Briefly, 5 mg each of curcumin and artesunate was added to an organic phase containing homogenous solution of 50 mg PLGA in 3.5 mL of dichloromethane and 0.5 mL of acetone to constitute 1:5 (drugs to PLGA) ratios. The organic phase was then added dropwise to 16 mL aqueous solution (2% PVA as emulsifier) with sonication at 30 W, 40% duty cycle for 3 min in ice cold water. The emulsion was continuously stirred for 6 h until all solvents completely evaporated. A colloidal suspension of the CA-PLGA nanoparticle was retained in the aqueous phase. The formulation was centrifuged at 16,000 × g for 15 min and this was followed by three washes. A 5% mannitol was added as cryoprotectant, and lyophilized to obtain a dry powder nanoformulation at −50°C.

### CA-PLGA nanoparticle size measurement and zeta potential

CA-PLGA nanoparticle diameter, zeta potential and polydispersity index (PDI) were measured by dynamic light scattering (DLS) method using Zetasizer Nano-ZS (Malvern Instruments, UK) as previously described (Busari et al., 2017; Dauda et al., 2017). The size and PDI of CA-PLGA nanoformulation were determined from nanoparticle suspension of appropriate amount of the drug loaded nanoparticles dispersed in MQ water in a clear disposable sizing cuvette. Zeta potential was analyzed by a clear zeta cell. Readings were taken in triplicate and results were expressed as mean ± SD.

### X-ray diffraction (XRD) and differential scanning calorimetry (DSC) of CA-PLGA nanoparticle

CA-PLGA nanoparticle was subjected to X-ray diffraction (XRD) analysis using an X'Pert-PRO multipurpose X-ray diffractometer (PANalytical, Netherland). The CuKα radiation was generated at 45 kV and 40 mA in the diffraction angle 2θ range of 5–40°. Differential scanning calorimetry (DSC) study to characterize the physical state of CA-PLGA nanoparticle was obtained using DSC PerkinElmer Pyris 1 (USA). The DSC cell was purged by dry nitrogen gas at a flow rate of 40 mL/min. A small quantity of the formulation was sealed in a standard aluminum pan with a lid and heated at a rate of 5°C/min from 50 to 300°C (Busari et al., 2017).

### Drug entrapment and encapsulation efficiency

A total of 10 mg lyophilized CA-PLGA nanoparticle was dissolved in 1 mL acetonitrile. The homogenous solution was evaporated for 9–10 h at 50°C using heater (CH-100, Biosan Ltd.). A resuspension of residue was carried out with 500 μL methanol. The mixture was vortexed and centrifuged at 13,000 × g for 20 min. Five hundred microliters of supernatant was collected and stored. The process was repeated with 500 μL of acetonitrile (Busari et al., 2017; Dauda et al., 2017).

A stock solution (100 μg/mL) of curcumin-artesunate was prepared from dissolution of 2.5 mg of each drug in methanol (5 mL). The drug concentration of non-entrapped free drugs was determined using UV-vis spectrophotometer (Ultrospec® 2100 *pro*, Amershan Biosciences) at isobestic wavelength 278 nm according to the method described by Birajdar et al. (2011). Also absorbance of free artesunate and curcumin was measured separately at 222 and 446 nm, respectively. Curcumin and artesunate contents in the CA-PLGA nanoparticle were estimated using the formula described by Birajdar et al. (2011).

$$\begin{array}{l} {\text{C}}_{1}=\frac{{\text{Q}}_{\text{o}}\;-\;{\text{Q}}_{2}\;\times \text{ A}}{{\text{Q}}_{1}\;-\;{\text{Q}}_{2}\;\times \;{\text{a}}_{1}}\\ {\text{C}}_{2}=\frac{{\text{Q}}_{\text{o}}\;-\;{\text{Q}}_{1}\;\times \text{ A}}{{\text{Q}}_{2}\;-\;{\text{Q}}_{1}\;\times \;{\text{a}}_{2}}\\ {\text{C}}_{1}=\text{Artesunate contents in CA}-\text{PLGA}\\ {\text{C}}_{2}=\text{Curcumin contents in CA}-\text{PLGA}\\ {\text{Q}}_{0}=\frac{\text{Absorptivity of CA}-\text{PLGA at }222\text{ nm}}{\text{Absorptivity of CA}-\text{PLGA at }278\text{ nm}}\\ {\text{Q}}_{1}=\frac{\text{Absorptivity of artesunate at }222\text{ nm}}{\text{Absorptivity of cartesunate at }278\text{ nm}}\\ {\text{Q}}_{2}=\frac{\text{Absorptivity of curcumin at }446\text{ nm}}{\text{Absorptivity of curcumin at }278\text{ nm}} \end{array}$$

In the above equation A was absorbance of sample at isoabsorptive point and a_1_ and a_2_ were absorptivity values of artesunate and curcumin, respectively, at isoabsorptivity point.

The calibration plot of non-entrapped drugs was obtained at concentrations 5–70 μg/mL. The supernatant containing CA-PLGA nanoparticle was also analyzed spectrophotometrically. Then the encapsulation efficiency of the formulation was estimated (Oyeyemi et al., 2018).

### *In vitro* release kinetics of drug-entrapped nanoparticle

Drug release study was carried out by suspending 10 mg of lyophilized CA-PLGA nanoparticle in 10 mL of phosphate buffered saline (PBS) pH 7.4, as the release medium. The mixture was incubated at 37°C in rotary shaker (New Brunswick Scientific, USA) at 200 × g. At predetermined time interval, the sample was centrifuged at 16,000 × g for 10 min after which 1 mL of supernatant was collected and then replaced with equal amount of fresh PBS.

A stock solution (1,000 μg/mL) of free curcumin and artesunate (2.5 mg each) was prepared in 5 mL methanol. Using PBS pH 7.4 as diluent, the working standard concentrations were prepared from the stock solution. The UV-absorbance was recorded at 278 nm. The UV-absorbance analysis of the supernatant from CA-PLGA nanoparticle at different time intervals was also carried out. The *in vitro* drug release from CA-PLGA nanoformulation was estimated from the standard plot obtained from UV-absorbance analysis of the non-entrapped drugs (Oyeyemi et al., 2018).

### Experimental animals and parasite strain

All experiments involving the use of laboratory animals adhered to the Principles of Laboratory Animal Care (NIH publication #85-23, revised in 1985). Approval for the study was granted by Animal Care Use and Research Ethics Committee (ACUREC) of the University of Ibadan, Nigeria (UI-ACUREC/17/0067). Male albino mice and *P. berghei* NK-65 were obtained from the Animal House Center of the Department of Pharmacology and the Institute of Advanced Medical Research and Training (IAMRAT), University of Ibadan, Nigeria, respectively.

### Peters' 4-day *Plasmodium berghei* suppressive test

Twenty male albino mice (5–6 weeks old) constituting five mice per experimental group were used in a Peters' 4-day suppressive test. Animals were maintained in standard pathogen-free conditions and fed *ad libitum*. *P. berghei* infected red blood cells obtained from a donor male mouse was adjusted to 1 × 10^7^ pEry/mL in physiological saline. Mice were infected intraperitoneal (ip) with 0.2 mL aliquot of parasites suspension (Busari et al., 2017). The CA-PLGA nanoparticle was reconstituted in 10% Tween 80 at different concentrations (5 and 10 mg/kg). The mice were administered orally with 0.2 mL of the nanoformulation suspensions 2-h post-infection. The positive and negative control groups received oral dose of 4 mg/kg chloroquine and artesunate (1:1), and 0.2 mL of the vehicle, respectively. Chloroquine and artesunate combined therapy was used based on a report of better efficacy in the form (Drakeley et al., 2004) and a result of a preliminary evaluation which confirmed its superior antiplasmodial activity compared with artesunate monotherapy. Treatments were further administered for 3 days. At day 5 and 8 post infection, blood smears were made from the tail vein, Giemsa-stained, and examined microscopically in immersion oil (Cheesbrough, 1998). Parasitized red blood cells were counted per total erythrocytes in four fields and the percentage parasite suppression was calculated.

### Hematological and hepatic toxicity assays

Acclimatized albino rats (94–105 g) were given oral doses of CA-PLGA nanoparticle at varying concentration (5 and 10 mg/kg), 4 mg/kg of positive control and vehicle as the negative control for 4 days. Blood samples (2 mL) obtained through retro-orbital puncture was collected into EDTA tubes and hematological parameters were determined by standard procedures. Plasma from centrifuged blood was assayed for liver enzymes determination using Randox kit following manufacturer's procedures.

### Statistical analysis

Data were analyzed using GraphPad Prism 6 (GraphPad Software, Inc., La Jolla, CA, United States). Two-way analyses of variance (ANOVA) and Tukey's multiple comparison tests were used to test for significant differences. Statistical significance was determined at *p* < 0.05.

## Results

### Characterization and *in vitro* release of nanotized CA-PLGA

**Figure 1:**
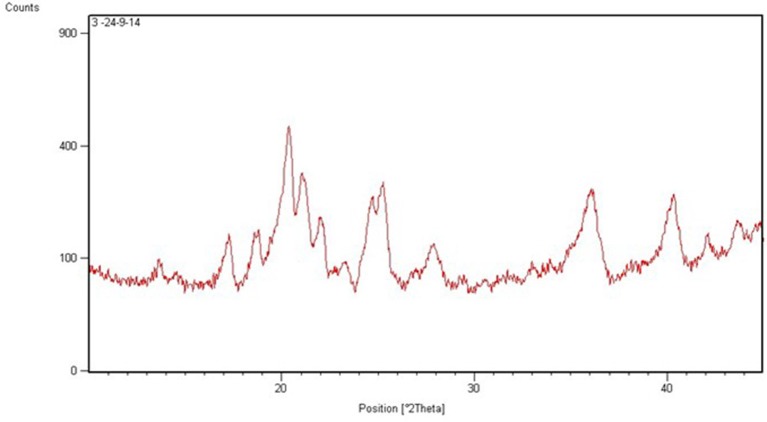
XRD spectra of CA-PLGA nanoparticle.

**Figure 2:**
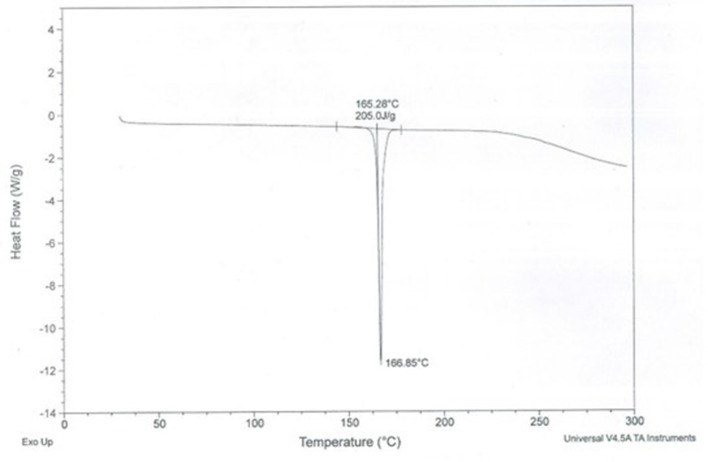
DSC thermogram of CA-PLGA nanoparticle.

**Figure 3:**
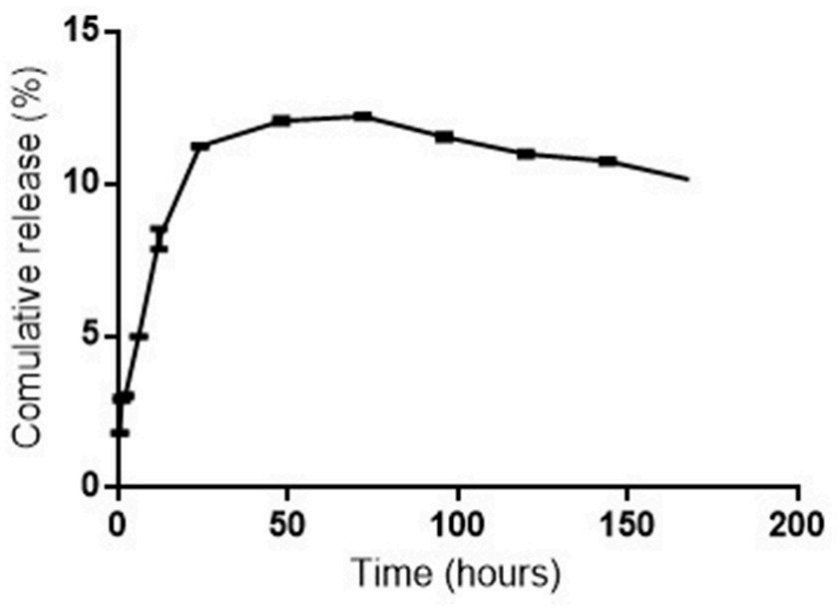
Cumulative *in vitro* release of curcumin and artesunate from CA-PLGA nanoparticle.

The size of the formulated CA-PLGA nanoparticle was 251.1 ± 12.6 nm. The PDI and zeta potential of the nanoparticle were 0.141 ± 0.06 and −19.1 ± 4.9 mV, respectively. The total curcumin-artesunate co-entrapped in the nanoformulation was 22.3 ± 0.4%. The individual curcumin and artesunate contents determined by spectrophotometric method were 0.906 and 0.114 mg/mL, respectively. The Powder X-ray diffraction (P-XRD) diffractogram for CA-PLGA is presented in Figure 1. The nanotized CA-PLGA characteristic intensity peaks were visible at 13.6, 17.3, 18.8, 20.4, 21.0, 22.0, 25.3, 27.8, 36.0, 40.4, 42.1, and 43.8° 2θ. The DSC thermogram of PLGA entrapped drugs is shown in Figure 2. The endothermic melting peak of nanotized CA-PLGA was 166.9°C. A biphasic drug release pattern was observed with an initial burst of about 4.7% of the total release within 1 h followed by a sustained curcumin-artesunate release (Figure 3).

### Antiplasmodial activity of nanotized CA-PLGA

**Figure 4:**
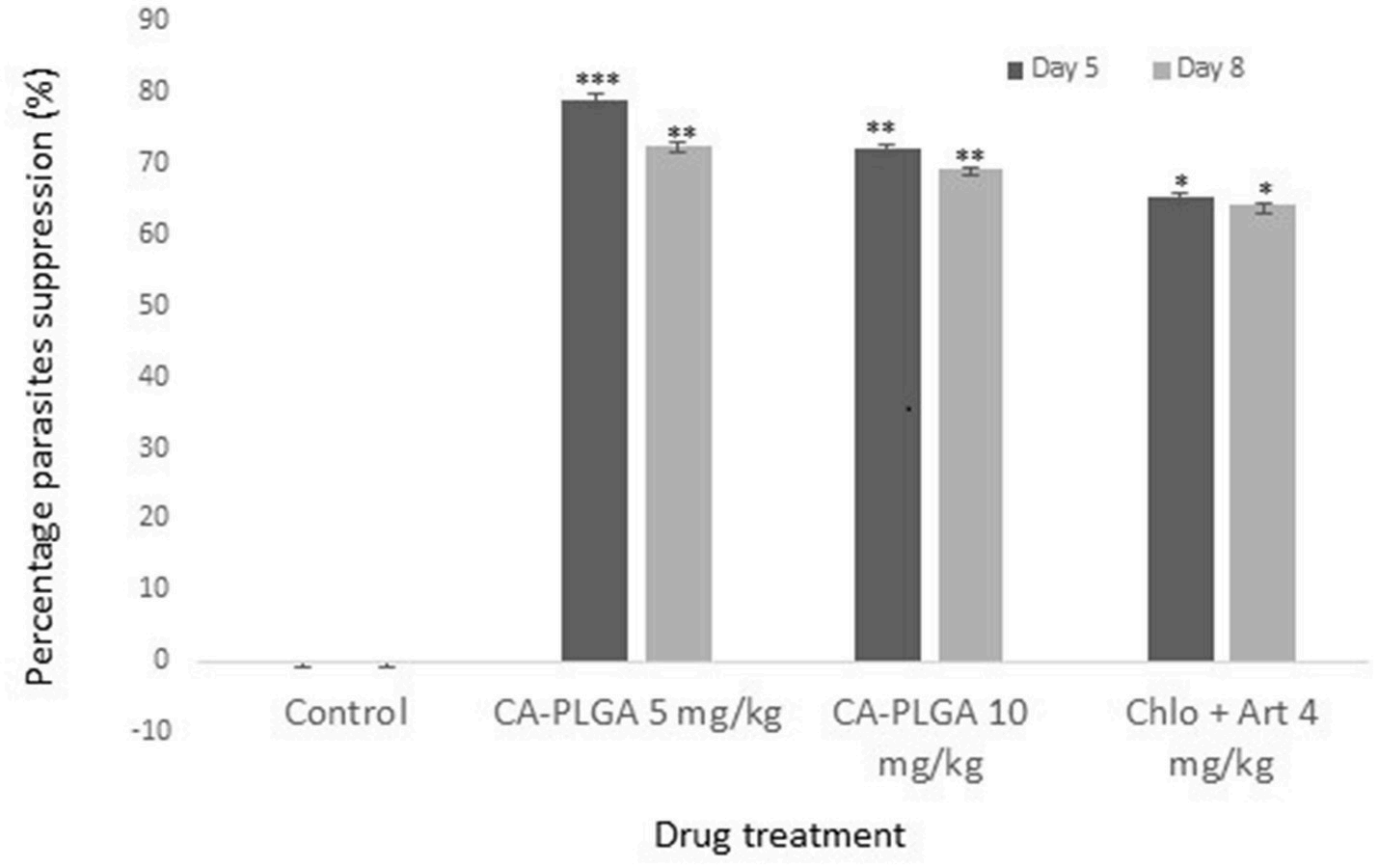
Parasitemia suppression (%) of CA-PLGA nanoparticle relative to concentration and duration of treatment. ^**^ was significantly higher than ^*^.

The *P. berghei* suppression rate was significantly higher at day 5 (79.0%) than at day 8 (72.5%) in 5 mg/kg CA-PLGA mice treated groups. Efficacies of nanotized drugs at 5 and 10 mg/kg at day 5 and day 8 were significantly higher than it was in the positive control group (*p* < 0.05). Efficacy however, was significantly higher in lower dose (5 mg/kg) of nanotized drug than in the higher dose (10 mg/kg) group at day 5 (*p* < 0.05). At day 8, there was no significant difference in parasite suppression in mice treated with 5 mg/kg (72.5%) and 10 mg/kg (69.3%) nanotized drugs (*p* > 0.05) (Figure 4). There was no parasite suppression in the negative control group.

### Survival of mice and toxicity of nanotized CA-PLGA

**Figure 5:**
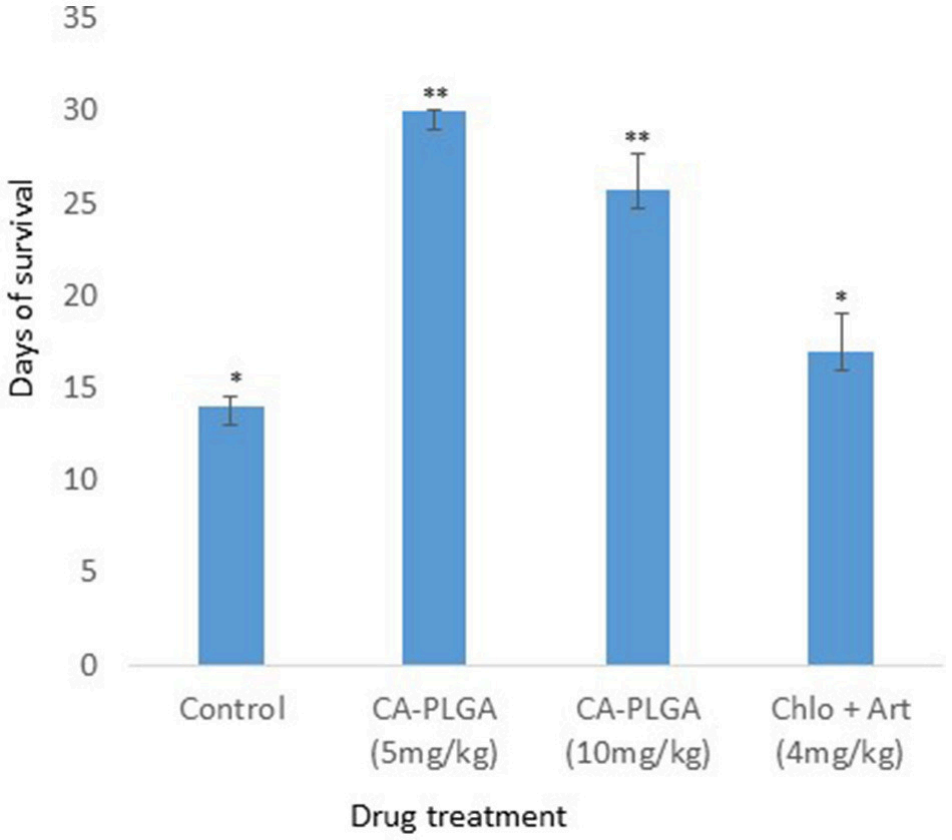
Survival of mice after drug administration. ^**^ was significantly higher than ^*^.

The survival rate of mice exposed to nanotized CA-PLGA at 5 and 10 mg/kg was significantly higher than in the negative and positive controls (*p* < 0.05) (Figure 5). The variation in mean liver enzymes' concentrations relative to CA-PLGA nanoparticle doses is shown in Table 1. Aspartate aminotransferase (AST) production was significantly lower in mice that were administered 5 mg/kg nanotized CA-PLGA (42.0 ± 0.0 U/L) and 10 mg/kg CA-PLGA nanoparticle (39.5 ± 3.5 U/L) compared with the negative control (45.0 ± 4.0 U/L) (*p* < 0.05). AST (42.0 ± 0.0 U/L) and ALP (80.0 ± 3.0 U/L) in 5 mg/kg CA-PLGA exposed mice were significantly higher than the values recorded (AST, 39.5 ± 3.5; ALP, 49.5 ± 17.5 U/L) in the 10 mg/kg CA-PLGA exposed mice (*p* < 0.05). ALP (114.0 ± 11.0 U/L) in the positive control was significantly higher than the values recorded in the CA-PLGA nanotized treated groups and the negative control (*p* < 0.05).

**Table 1:** Hepatic toxicity of CA-PLGA nanoparticle.

**Liver enzymes (U/L)**	**CA-PLGA (5 mg/kg)**	**CA-PLGA (10 mg/kg)**	**Chlo+Art (4 mg/kg)**	**Negative control**
AST	42.0 ± 0.0^a^	39.5 ± 3.5^b^	36.5 ± 0.5^d^	45.0 ± 4.0^c^
ALT	31.5 ± 0.5^a^	29.5 ± 2.5^a^	27.0 ± 2.0^b^	32.0 ± 2.0^a^
ALP	80.0 ± 3.0^a^	49.5 ± 17.5^b^	114.0 ± 11.0^c^	105.0 ± 24.0^d^

*Similar superscript letters denote there are no significant differences while different alphabets denotes there are significant differences. AST, Aspartate aminotransferase; ALT, Alanine aminotransferase; ALP, Alkaline phosphatase*.

There were no significant differences in the mean hematological parameters in the different nanotized CA-PLGA doses' groups relative to the negative control (*p* > 0.05). The platelet counts however, were significantly raised in the positive control group (4 mg/kg Chlo+Art) (Table 2).

**Table 2:** Effects of CA-PLGA nanoparticle concentrations on hematological parameters.

		**Drugs**		
**Blood parameters**	**Control**	**CA-PLGA**	**CA-PLGA**	**Chlo+Art**
		**(5 mg/kg)**	**(10 mg/kg)**	**(4 mg/kg)**
PCV (%)	37.0 ± 1.0^a^	35.0 ± 1.0^a^	36.5 ± 0.5^a^	38.5 ± 0.5^a^
Hb (g/dL)	12.1 ± 0.6^a^	11.3 ± 0.1^a^	12.1 ± 0.3^a^	12.7 ± 0.1^a^
RBC (× 10^6^/cm^3^)	6.3 ± 0.1^a^	5.7 ± 0.5^a^	6.3 ± 0.07^a^	6.4 ± 0.1^a^
WBC (× 10^3^/cm^3^)	8.3 ± 2.3^a^	4.6 ± 0.5^a^	6.7 ± 1.7^a^	9.9 ± 3.2^a^
Platelets(× 10^3^/cm^3^)	110.5 ± 41.0^a^	104.0 ± 44.0^a^	119.5 ± 0.6^a^	217.5 ± 7.5^b^
Lymphocytes (%)	66.5 ± 4.5^a^	64.5 ± 1.5^a^	67.0 ± 2.0^a^	63.0 ± 2.0^a^
Neutrophils (%)	30.5 ± 3.5^a^	32.5 ± 2.5^a^	29.5 ± 2.5^a^	34.0 ± 1.0^a^
Monocytes (%)	1.5 ± 0.5^a^	2.0 ± 1.0^a^	1.0 ± 0.0^a^	2.0 ± 1.0^a^
Eosinophils (%)	1.5 ± 1.5^a^	1.0 ± 0.0^a^	2.5 ± 0.5^a^	1.0 ± 0.0^a^

*Similar superscript letters denote no significant difference while different letters denote a significant difference. PCV, Packed cell volume; Hb, Hemoglobin; RBC, Red blood cell; WBC, White blood cells*.

## Discussion

This study employed a simple method of dual entrapment of curcumin and artesunate in a nanoformulation for improved malaria treatment. A biodegradable and biocompatible PLGA nanomaterial was used for the encapsulation in order to increase the bioavailability of the two parent compounds. Although the encapsulation efficiency was low, the nanoparticulate size and zeta potential of the formulation remained within the acceptable range (Cong et al., 2010). The low PDI is desirable and will facilitate the monodispersibility of the nanotized CA-PLGA (Luz et al., 2012). The 4.7% initial burst release of entrapped curcumin and artesunate followed by a controlled release of the drugs will offer the formulation an early therapeutic advantage. Decrease of curve at about 75 h could be due to a slight degradation of drug at the later time points or might be due to polymer interference. The XRD spectra showed the crystallinity nature of the formulation and this may be due to the use of 5% mannitol as cryoprotectant during freeze drying. This effect might have been more pronounced owning to the fairly larger nanoparticulate size of our formulation (Panyan et al., 2004). The nanotized CA-PLGA is physically stable due to absence of decomposition exotherm in the formulation (Chadha et al., 2012).

With evidences of drug resistance to common malaria monotherapy and ACTs, there are needs for new alternatives with better efficacy. We have recently explored the antimalarial potency of PLGA single encapsulation of artesunate and curcumin and the studies showed improved activities against the drugs' pure forms (Busari et al., 2017; Dauda et al., 2017). Overall, our CA-PLGA formulation showed an impressive potency with more than 10-30% increase in parasite suppression when compared with free curcumin, free artesunate, or the nanotized forms of each of the pure drugs as reported in our previous studies (Busari et al., 2017; Dauda et al., 2017). While curcumin and artesunate have been recently utilized in malaria combined therapy in murine model (Desai et al., 2015), this study was the first to evaluate their combined antimalarial potential in a delivery system. One closely related study however, investigated the antimalarial activity of free artemisinin plus curcumin liposomal nanoparticle (Isacchi et al., 2012). Besides the cost effectiveness of turmeric curcumin, its ability to offer protection against artesunate-induced oxidative stress (Desai et al., 2015) poses further advantage in their combination in a delivery system.

A preliminary unreported finding showed an unfavorable pharmacodynamics interaction in combined free form of curcumin and artesunate. Similar finding was observed in curcumin/piperine/artemisinin combination in a previous report (Neto et al., 2013). While a favorable antimalarial potential of combined form of free curcumin and artemisinin was recorded against the blood stage of *P. berghei*, the combined therapy was unable to attack the liver and spleen parasite schizonts (Vathsala et al., 2012) thus favoring recrudescence. So, the delivery system of the two drugs may help to overcome this challenge and better harness their antimalarial potential.

The better antimalarial activities of nanotized CA-PLGA observed at 5 mg/kg was similar to our recent study on curcumin-PLGA entrapped nanoparticle (Busari et al., 2017). Although the reason for this is not yet ascertained, it could offer a therapeutic advantage as a low dose nanotized drug can achieve a similar or better antiplasmodial effect. This is usually in deference to common observation with conventional free drugs where activities are oftentimes expected to be dose dependent. Prolonged circulation of nanotized drugs and improved bioavailability and absorption influenced by the small particle size have been suggested to be responsible for their improved activities (Busari et al., 2017). Improvement in antiplasmodial activities in low dose nanotized drug also correlated with increase in the mice life span. The reduction in the mice life span observed in the control group was expected as the animal would suffer more from the morbid effects of parasites' infection in the absence of treatment. Curcumin and artesunate were released for more than 1 week in the *in vitro* release profile; a property which may improve the antimalarial activity through prolonged activities. The ability of nanoparticle to deliver encapsulated drugs to target organs or tissues may also avail the formulation the ability to reach the parasite schizonts in the liver or spleen.

It was evident in our study that the nanotized CA-PLGA formulation was non-toxic as most of the enzyme indicators of hepatoxicity were either significantly lower or showing no significant differences in comparison with the negative control. The safety of the nanoparticle can also be observed in the results obtained from the hematological profiles. The seemingly desirable liver enzymes profiles observed in the positive control group needs further studies. The potential of curcumin to suppress the ability of artesunate to generate reactive oxygen species (ROS) from its endoperoxide bond leading to production of lipid peroxide which is toxic to host cells had earlier been reported (Robert et al., 2002; Desai et al., 2015). The delivery system which ensures controlled release of drugs could have further aided their positive synergistic interaction leading to the nontoxicity of the nanotized CA-PLGA.

The nanotized CA-PLGA formulation was novel, simple to develop and nontoxic. The drug was very active against malaria parasite at a very low concentration and therefore can offer a novel therapeutic approach for malaria treatment. Further optimisation to obtain a higher drugs entrapment efficiency and studies on mechanisms of action of the formulation are recommended.

## Author contributions

OO: Conception, design, laboratory studies, data analysis, and manuscript drafting; OM: Conception and manuscript revision; FA: Conception, design, laboratory studies, manuscript revision; KD and ZB: Laboratory studies; JM: Design, laboratory studies, manuscript revision; AP: Design, materials contribution.

### Conflict of interest statement

The authors declare that the research was conducted in the absence of any commercial or financial relationships that could be construed as a potential conflict of interest.
